# Gamified Physical Education and Cognitive Performance Among Chinese Secondary School Students: Cross-Sectional Moderation Mediation Study

**DOI:** 10.2196/81086

**Published:** 2026-02-02

**Authors:** Jingtong Zhang, Sae-Sook Oh, Yaru Xu

**Affiliations:** 1 Department of Physical Education Kyonggi University Suwon-si Republic of Korea; 2 School of Health Sciences Universiti Sains Malaysia Kubang Kerian, Kelantan Malaysia

**Keywords:** gamified physical education, cognitive performance, intrinsic motivation, AI-based feedback, teacher support, peer collaboration, China, artificial intelligence

## Abstract

**Background:**

Educators are exploring new methods to educate beyond the classroom as global concerns about students’ cognitive, emotional, and social well-being grow. Physical education (PE) has been demonstrated to boost cognitive and psychological outcomes in several studies. Most research has neglected the benefits of gamification and artificial intelligence (AI)–based feedback in PE, focusing instead on conventional PE formats. The impacts of technologically enhanced PE settings on students’ cognitive performance through feedback and reward mechanisms remain understudied.

**Objective:**

This study aimed to investigate how intrinsic motivation and AI-based feedback moderated the effects of gamified PE on students’ cognitive performance.

**Methods:**

The study used a cross-sectional design. In Beijing, Shanghai, Chengdu, and Guangzhou, a total of 1029 public high school students completed a standardized questionnaire. Students in secondary school (male: n=490, 47.6% and female: n=539, 52.4%) aged 10-18 years, were recruited from urban, suburban, and rural locales. Participants were sourced from public, private, and semigovernment schools, reflecting a range of academic achievement levels and access to technology. Students participating in standard PE sessions were included, whereas those with medical conditions that restricted physical exercise were excluded. Data were gathered via standardized questionnaires during designated PE sessions. Gamified PE, cognitive performance, intrinsic motivation, teacher support, collaboration, and AI feedback were examined using standardized instruments. Trained facilitators helped younger participants understand and follow ethical norms. The study used maximum likelihood estimation for structural equation modeling. Bootstrapping was used to analyze mediation and moderation effects at a 5% significance level (α=.05).

**Results:**

According to structural equation modeling, gamified PE highly predicts cognitive performance (β=.34; *P*<.001). Other significant factors were teacher support (β=.31; *P*<.001), physical exercise enjoyment (β=.28; *P*<.001), and teamwork (β=.26; *P*<.001). AI-based feedback strengthened the link between gamified PE and cognitive performance under moderation analysis (β=.18; *P*<.001). Mediation analysis indicated that intrinsic motivation partially mediated the relationship, resulting in a significant indirect effect (β=.21, 95% CI 0.12-0.31; SE=0.05).

**Conclusions:**

This research integrates gamified PE with AI-based feedback mechanisms to evaluate students’ cognitive outcomes, a domain that has been rarely investigated experimentally. This study highlights the combined effect of intrinsic motivation and AI-generated feedback in a technology-enhanced PE context, in contrast to previous research that primarily focuses on traditional PE methods or isolated gamification elements. The findings enhance the field by demonstrating that student-centered, feedback-rich PE environments may improve cognitive abilities through social interaction, enjoyment, and instructor support. AI-assisted, gamified PE programs may enhance learning outcomes and academic performance among secondary school students.

## Introduction

### Background of Research

In the 21st century, multimodal techniques that integrate physical, cognitive, emotional, and technical components have become more important in education [1]. Contemporary research suggests that physical and intellectual education must be integrated, even though traditional schools generally divide them [2,3]. Physical education (PE) can change children’s lives by enhancing their emotional, cognitive, and academic performance [4]. Concerns about student inactivity and cognitive overload have led to the development of gamified PE, an innovative teaching approach. This strategy uses game-design principles to make exercise more enjoyable, goal-oriented, and cognitively demanding [5]. Gaming methods combined with physical exercise have been shown to enhance motivation, teamwork, and self-control—essential for both mental and physical well-being [6].

Gamification incorporates features such as points, competition, prizes, badges, levels, and leaderboards into nongame contexts to motivate and engage users. Gamification, which originated in marketing and business, is now being used in classrooms to engage students [7]. This type of PE transforms static exercises into game-like cooperative learning settings where students work together to achieve goals. Research suggests that gamified methods improve students’ engagement, enjoyment, and academic success [8]. Teenagers often find PE uninteresting or irrelevant; therefore, these strategies have been effective in capturing their attention. Gamified PE promotes active participation and cognitive engagement by allowing students to make choices, solve problems, and reflect on outcomes in real time [9].

Cognitive performance, encompassing attention, memory, reasoning, and problem-solving, has been extensively studied in educational psychology about physical well-being [10,11]. Regular exercise improves working memory, executive function, and information processing speed. Neurobiological mechanisms, including increased cerebral blood flow, reduced cortisol levels, and the release of brain-derived neurotrophic factor, may explain this connection [12]. Gamified PE may directly stimulate cognitive brain networks through rule-following, decision-making, instruction, memory, and strategy adjustment [13]. China’s rapidly changing educational system is a fascinating environment for studying these dynamics. China has put student well-being alongside academic success in recent years to address excessive academic pressure, mental health issues, and physical inactivity in school-age children and adolescents [14]. National educational reforms aim to provide students with a “quality education” that nurtures their emotional, physical, and cognitive potential [15]. Chinese schools are experimenting with gamified teaching, notably in PE, to achieve this goal [16]. Despite well-defined policy aims, empirical research on the implementation processes and outcomes of these innovations is lacking. Understanding the impact of gamified PE on cognitive development and academic achievement in Chinese children requires considering psychological factors, such as motivation, and external factors, such as technology use.

Intrinsic motivation links gamified PE to cognitive performance. This is the desire to do something because it is fun, fascinating, or valuable. Ryan and Deci’s [17] self-determination theory states that intrinsic motivation is driven by autonomy, competence, and relatedness. Gamified situations provide autonomy, competence, and relatedness. Gamified aspects in PE may help students understand and connect with physical exercise by making repetitive tasks more interesting [18]. Intrinsic motivation enables students to self-regulate their participation, thereby improving cognitive functioning and task performance [19]. The mediating role of intrinsic motivation is crucial in understanding how gamified PE may impact cognitive outcomes. Gamified PE may be enhanced or mitigated by technology, particularly artificial intelligence (AI)–powered feedback systems. Many progressive schools worldwide use smart wearables, mobility trackers, and digital dashboards, with AI being increasingly integrated [20,21]. These technologies enhance training efficiency by offering adaptive learning environments, personalized feedback, and real-time performance monitoring. AI may transform PE by enabling real-time monitoring of students’ and teachers’ efficiency, speed, endurance, and progress [22]. By acknowledging effort, fostering reflection, and driving self-improvement, such technologies may increase student engagement when gamified. Student receptivity to technology, teacher implementation skills, and institutional resources can all impact their effectiveness. Thus, AI-based feedback may moderate the relationship between gamified PE and cognitive performance sufficiently to change its direction.

In addition to these core concepts, contextual considerations should also be addressed. In China, a collectivist culture that values group cohesiveness and cooperative learning, peer collaboration is important. Gamified PE involves collaborative, communicative, and goal-oriented teamwork [23]. This may improve relationships and higher-order thinking via collaborative problem-solving and strategy building. Teacher support also affects student engagement in gamified contexts. When teachers model healthy exercise habits, provide constructive feedback, and encourage autonomy, they may motivate and engage students. Instructor behavior and gamified content delivery are crucial factors in assessing the effectiveness of an intervention [24]. Another key component in China’s educational background is the variety of school settings, from public to private and urban to rural. The accessibility of gamification and AI technologies may vary. In Shanghai and Beijing, schools have smart classrooms and instructors who use technology to make learning an engaging experience [25]. Rural or low-resource schools often struggle with outdated technology, a shortage of qualified personnel, and resistance to innovative teaching methods. Demographics and institutional characteristics may help explain differences in student cognitive performance. Confucian values, including diligence, self-control, and academic achievement, also shape Chinese educational perspectives [26]. These principles influence students’ perceptions of gamification and other innovative teaching methods. Gamified PE may be invigorating for some students but too limited for others. Thus, cultural perception is a subtle yet powerful factor that may influence new teaching approaches [27]. To achieve cognitive and motivational advantages, gamified PE may need culturally relevant values and communication strategies.

This study should be viewed from the perspective of modern educational research, which increasingly promotes interdisciplinary collaboration. Motivation theory, PE, educational technology, and cognitive science must collaborate to understand student learning. Gamified PE combines movement studies, behavioral psychology, and technology. These cross-domain interactions are seldom studied, particularly in China’s sociocultural context. Gamification in academic fields such as mathematics or language learning has garnered the greatest attention [28], whereas most research has focused on the cognitive advantages of physical exercise [29]. Technical, motivational, and cognitive studies on gamified PE as a core intervention are lacking.

This study is theoretically and practically important for Chinese educational innovation and student growth. Gamifying PE programs can improve children’s health and engagement, a trend that is becoming increasingly significant in schools worldwide [30]. Gamified PE may increase physical activity and cognitive function in China, where academic achievement often takes precedence over emotional and physical growth [31]. Studying the mediating role of intrinsic motivation reveals the psychological underpinnings behind student learning and engagement. While educational technology shapes learning settings, AI-based feedback serves as a moderating component that addresses this tendency. This study’s results contribute to the global discussion on educational reform, technology-enhanced learning, and student motivation, as it is one of the first to systematically analyze these factors within the Chinese secondary school system. New technologies help school administrators, policymakers, and educators develop more engaging, productive, and intellectually stimulating PE programs.

Today’s educational studies must grasp how PE, cognitive development, and motivational psychology interact, particularly in rapidly modernizing nations such as China. Given the growing use of gamified methods and AI in education, research on their influence on key learning outcomes is urgently needed. Over the past 20 years, research on the cognitive benefits of exercise has evolved. Still, the impact of 2 modern pedagogical innovations, namely gamification and AI-based feedback, on this area remains unknown. This study emphasizes gamified PE, intrinsic motivation, AI-based feedback, and cognitive performance. The 3subsections of the review conclude with a hypothesis to guide empirical research.

### Gamified PE and Physical Activity Enjoyment With Cognitive Performance

Several studies have demonstrated that gamified PE enhances children’s learning and brain development [30,32]. Rule-based, competitive, and strategic games boost students’ attention, engagement, and decision-making in gamified PE [33]. Gamified learning activities have been shown to improve task completion and working memory. Gamified physical activities compel students to solve problems, devise strategies, and assess their progress in real time, which promotes higher-order thinking. These benefits stem from PE games and competitions that keep children moving and engaged in critical-thinking activities [34]. Gamified PE lessons improved content retention and self-regulated learning. It is widely recognized that exercise enhances cognitive function. Students who like exercising are more likely to persist with it. This aids brain growth and executive function [35,36]. A meta-analysis found that children who were happier while exercising performed better on cognitive flexibility and memory tests. Engaging and pleasant activities promote academic learning, critical dual-task performance, and prefrontal brain activity. Despite a lack of scientific data, gamification is gaining popularity in China as a teaching method [37,38]. Gamified PE treatments in Shanghai, China, have improved students’ memory and attention. Gamified PE increased mental rotation exercises and digit span recall. These results support the premise that engaging and interesting physical exercise promotes both physical and psychological health. Gamification’s novelty must be managed carefully to keep users interested and minimize cognitive fatigue [39,40].

### Peer Collaboration and Teacher Support to Cognitive Performance

Working together in class has long been shown to boost students’ social and cognitive development. Collaborative learning plays a key role in intellectual growth. Teamwork and collaboration in PE foster effective communication, critical analysis, and perspective-taking [41,42]. Students who worked together on physical activities performed better on reflective thinking and adaptation exams. Peer contact in PE improves strategic thinking and decision-making, particularly when participants must collaborate to solve challenges or reach a consensus [43,44]. Coordinated peer collaboration in PE enhances cognitive engagement. This was especially evident when students discussed approaches and reflected on their outcomes. Cognitive benefits have been observed in teaching games for understanding models, which rely heavily on peer interactions [45,46]. These models improve foresight, planning, and problem-solving. Collectivist principles that promote group cohesiveness and shared accomplishment make peer collaboration meaningful in Chinese education. Chinese students who took PE programs with a partner performed better on executive functioning assessments. Peer collaboration and teacher support help influence students’ cognitive involvement [47]. Teachers who foster autonomy and provide clear feedback help pupils build intrinsic motivation and learning perseverance. PE teachers’ primary duties include engaging students and making the classroom exciting. Scaffolding, encouragement, and feedback improve cognitive performance [48,49]. Physical ability and academic success were higher in Chinese students who were more encouraged by their PE teachers. Formative assessments and motivational signals reduced cognitive load and increased self-reflection in PE students [50]. Gamified PE highlighted the need for psychologically safe spaces where children can experiment, fail, and learn, which is essential for cognitive development [51]. Instructors remain crucial to PE courses that use AI to support students in understanding digital feedback and to foster metacognition [52]. These findings suggest that students need to collaborate and have their teachers’ support to increase cognitive performance, particularly in dynamic and game-based learning environments.

### Mediating Role of Intrinsic Motivation and Moderating Effect of AI-Based Feedback

Intrinsic motivation, which is the drive to do something for its own sake, has been linked to scholastic and cognitive success. Gamified learning settings make students feel more connected, competent, and independent [53]. Motivation to study enhances students’ use of deeper cognitive techniques, sustained attention, and effort. Genuinely driven PE students performed better and persevered longer in motor learning tasks. Enjoyment and perceived competence during PE influenced the relationship between PE involvement and academic success [54,55]. Inner motivation mediated the relationship between physical activity and math performance in Chinese middle schools. A recent study suggests that gamified PE motivates students to enhance their executive functioning and facilitate learning transfer [56,57]. These findings support the cognitive benefits of gamified PE, including increased intrinsic motivation, perceived competence, and a sense of control. In contrast, AI has enhanced PE by providing students with real-time performance statistics. Smart wristbands equipped with AI-powered motion sensors facilitate reflective thinking and personalized learning. AI-based feedback enhanced students’ metacognitive awareness and adaptive learning strategies [58,59]. This input may reinforce learning loops, give immediate incentives, and drive persistent participation in gamified contexts. Students who received AI-enhanced PE courses performed better on spatial thinking and problem-solving examinations than those who received conventional instructor feedback. AI’s moderating influence is not always positive. Students may become excessively dependent on external validation or overwhelmed by AI technology, depending on deployment and preparation [60,61]. Thus, its effectiveness may vary depending on the situation. AI-enhanced PE pilot programs in China have shown promising outcomes, with improved engagement and physical literacy [62]; however, concerns remain regarding unequal access and teacher training. When paired with AI-based feedback, gamified PE may improve cognitive function, particularly depending on the quality of feedback, engagement, and customization.

### Research Gaps and Contribution of the Study

There is growing evidence that PE is important for cognitive development; however, the impact of gamified PE interventions on cognitive outcomes remains unclear, particularly in the Chinese educational and cultural context. Exercise has been shown to improve attention, memory, and executive functioning in several studies [63,64]. These studies have primarily examined aerobic or endurance activities, rather than PE programs that use games to enhance these areas. Gkintoni et al [65] found that motor skill memorization in conventional PE improves mental agility, while gamified activities that require flexibility, strategy, and decision-making in the present engage students’ brains more effectively.

Another topic with limited research is the impact of PE on cognitive functioning and its underlying mechanisms. Self-determination theory has been applied in motivation research; however, few studies have examined intrinsic motivation as a moderator in gamified PE [66,67]. Sañudo et al [68] observed that gamification boosts physical activity, but their models do not account for the psychological relationship between intrinsic motivation, internal pleasure, and mental performance. To develop educational interventions that are both physically and psychologically beneficial, it is essential to understand the driving factors of gamified learning.

Research on the cognitive effects of gamified PE using AI-based feedback is seldom conducted. Although AI is becoming increasingly widespread in developing or transitional education systems, such as China’s, academics have given less attention to its applications in PE. AI research in education has focused on academic learning platforms or disregarded pedagogy. Zha et al [69] study shows that AI-enhanced feedback tools may assist students in learning PE technical skills, but they do not link them to cognitive progress. In addition, there is limited knowledge about how technological tools affect motivational psychology. AI feedback in gamified PE has not been studied to determine whether it enhances self-awareness and metacognitive processing or merely motivates.

The literature on physically interactive, game-based learning environments does not address multilevel classroom dynamics, including student-teacher collaboration and instructor support. The social aspect of PE is often overlooked in empirical models that combine gamification and cognitive findings. Iglesias and Fernandez-Rio [70] conducted a comparative study. Students may be more engaged in collaborative PE. The research does not examine how these interactions increase cognition or how teacher scaffolding affects it. Comprehensive frameworks must incorporate instructional strategies, motivating factors, technological upgrades, and student interaction patterns, which affect cognitive performance.

This study examines how gamified PE, physical activity enjoyment, peer cooperation, and teacher support impact students’ cognitive performance in China’s unique educational system, aiming to fill these complex gaps. This study integrates pedagogical and psychological perspectives to better understand how innovative PE methods can enhance cognitive performance. The research incorporates both technology and physical exercise, unlike previous investigations. The study uses intrinsic motivation as a mediating component to enhance the theoretical understanding of internal motivational processes in gamified scenarios. It also examines how AI-based feedback moderates the effects of digital technology on learning in interactive, collaborative classrooms. The study suggests that secondary school PE programs facilitate students’ cognitive and developmental progress by providing data to inform curriculum development, teacher professional development, and the effective use of educational technology. It can help Chinese and other school administrators develop balanced, tech-integrated, and student-centered PE curricula to achieve 21st-century learning goals.

Research has linked gamified PE with an intrinsic desire to exercise and improved cognitive function to form the following research hypotheses:

- H1: Gamified physical education and physical activity enjoyment have a significant positive effect on students’ cognitive performance.
- H2: Peer collaboration and teacher support have a significant positive effect on students’ cognitive performance.
- H3: Intrinsic motivation mediates, and AI-based feedback moderates, the relationship between gamified physical education and cognitive performance.

## Methods

### Theoretical Framework

This study is based on cognitive load theory (CLT) and self-determination theory (SDT), with contextual support from Constructivist Learning Theory. Deci and Ryan’s [71] self-determination theory explains how students’ sense of enjoyment in connectivity, autonomy, and competence motivates them to pursue diverse activities. SDT is helpful for this study because it shows how intrinsic motivation moderates the relationship between gamified PE and cognitive function. Gamification elements, such as points, challenges, levels, and social engagement, help PE programs fulfill students’ psychological needs and encourage enthusiastic participation. As gamified PE becomes more engaging and personalized, students’ cognitive engagement, memory, and problem-solving abilities are expected to improve [72]. CLT suggests that gamification and other instructional methods may increase working memory and decrease cognitive load, particularly when combined with real-time AI-based feedback [73]. Such feedback may help students focus, repair mistakes quickly, and learn more via adaptive responses by strengthening the gamified PE-cognition link. According to Constructivist Learning Theory, teachers can direct students’ exploration, social interaction, and feedback, while students can also help themselves through peer collaboration and instructor support [74]. Teamwork and teacher facilitation are highly effective in promoting engagement and the application of cognitive skills in PE.

**Figure 1:**
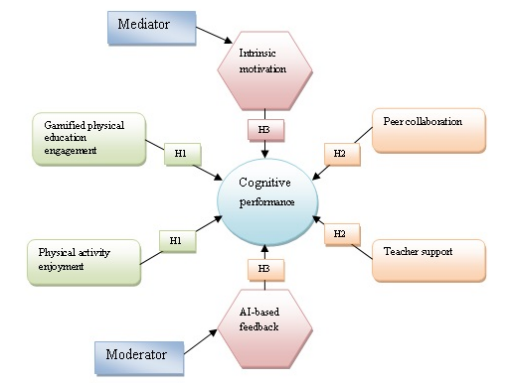
Conceptual framework showing hypothesized relationships among explanatory variables, mediator, moderator, and dependent variable (cognitive performance) with hypotheses H1–H3. Data from 1029 high school students in Beijing, Shanghai, Chengdu, and Guangzhou, 2025. Source: author’s work. AI: artificial intelligence.

These theoretical frameworks create the study’s expected variable relationships (refer to Figure 1).

The first hypothesis (H1) states that gamified PE and physical activity enjoyment will enhance students’ cognitive performance. Physically active classrooms boost students’ physical health, mental clarity, memory, and problem-solving skills. H2, which builds on H1, suggests that teacher and peer interaction enhances students’ cognitive performance by focusing on the social and pedagogical aspects of learning. These factors make the classroom a friendly yet challenging environment for students to learn through hands-on experiences, collaborate, and receive constructive feedback. H3, the technological and psychological components, reveals that intrinsic motivation mediates the relationship between gamified PE and cognitive performance and that AI-based feedback moderates this relationship. Intrinsically motivated students are more likely to focus, continue the course, and actively engage in learning using AI feedback systems, which improves outcomes. This paradigm encompasses motivation, instruction, technology, and cognition, and when combined, these elements demonstrate how gamified PE can support learning in today’s classrooms.

### Population, Sample, and Research Design

The study investigated the influence of intrinsic motivation and AI-based feedback on the relationship between gamified PE and students’ cognitive performance in China’s unique cultural and educational context. The research includes secondary school PE students from various regions in China. The sample included urban, suburban, and rural schools from Beijing, Shanghai, Chengdu, and Guangzhou municipalities. These regions were selected because they participate in national education innovation projects and have various degrees of classroom technology integration. The study used quantitative, cross-sectional surveys and structured questionnaires to obtain participant data. The 1029 completed and valid responses from 1175 surveys issued to 10- to 18-year-olds yielded an 87.6% response rate. Based on Cochran sample size calculation approach, which accounts for a 95% CI and a 3% margin of error, a sample of desired respondents was required. Multivariate statistical analysis, including structural equation modeling (SEM), can be reliably performed with the given sample.

### Demographic Profile

The study collected demographic data to understand the participants’ contextual profiles and ensure diversity across crucial parameters. High, moderate, and low achievers were categorized based on their academic achievement. The demographic component of the questionnaire inquired about school type (public, private, or semigovernment), location (urban, suburban, or rural), and access to technology (high, moderate, or low). The study cross-tabulated these factors to ensure consistency among student demographics and institutional settings.

### Measurement Scales

This survey included key factors from theoretical and empirical investigations (refer to Table 1). Cognitive performance was assessed using a Cognitive Functioning Scale item derived from the Woodcock-Johnson Tests. The item includes: “*My memory has improved due to gamified PE*.”A cognitive development study has validated this scale [75]. The first independent variable, gamified physical education engagement, was assessed using the game-based learning engagement scale. The item was *“I feel more engaged in gamified PE*,” followed by Hamari et al [76]. The second independent variable, physical activity enjoyment, was assessed using the physical activity enjoyment scale with a representative item, “*I feel energetic after physical activity,*” as suggested by Kendzierski and DeCarlo [77]. The third independent variable, examined by the collaborative learning scale, was “*Team-based PE helps me learn more,*” as used by Laal and Ghodsi [78] for instrumentation. Belmont et al [79] used the scale and representative items, including “*My teacher helps me reflect on progress,*” to score instructor support, the fourth independent variable, on the Teacher as Social Context Questionnaire–Autonomy Support subscale. The mediating variable, intrinsic motivation, was operationalized using the intrinsic motivation inventory-interest and enjoyment subscale. One key item is “*I feel accomplished after PE.*” Deci and Ryan’s [71] theoretical model and psychometric validation by McAuley et al [80] provided support for this instrument. AI-based feedback was measured using a sample item from the smart education technology–enhanced feedback scale, supporting Ifenthaler et al [81] and Caspari-Sadeghi et al [82]. The study used a 5-point Likert scale for all items, with 1 indicating “strongly disagree” and 5 indicating “strongly agree.”

**Table 1:** Measurement scales and sample items for studied variables, based on responses from 1029 high school students in 4 Chinese cities, 2025.

Constructs	Measurement scale	Sample item from questionnaire	Literature support
Cognitive performance (DV^a^)	Cognitive Functioning Scale (adapted from Woodcock-Johnson Tests)	I can concentrate better during lessons after gamified PE^b^.	[75]
Gamified physical education engagement (IV1^c^)	Game-Based Learning Engagement Scale	I enjoy gamified PE classes.	[76]
Physical activity enjoyment (IV2^d^)	Physical Activity Enjoyment Scale (PACES)	Physical activities are fun for me.	[77]
Peer collaboration (IV3^e^)	Collaborative Learning Scale	I work better in teams during PE.	[78]
Teacher support (IV4^f^)	Teacher as Social Context Questionnaire–Autonomy Support subscale	My PE teacher gives helpful feedback.	[79]
Intrinsic motivation (mediator)	Intrinsic Motivation Inventory (IMI): interest and enjoyment subscale	I participate in PE because I enjoy it.	[71,80]
^g^AI-based feedback (moderator)	Technology-Enhanced Feedback Scale	AI-based feedback helps me improve my physical skills.	[81,82]

^a^DV: dependent variable.

^b^PE: physical education.

^c^IV1: independent variable 1.

^d^IV2: independent variable 2.

^e^IV3: independent variable 3.

^f^IV4: independent variable 4.

^g^AI: artificial intelligence.

### Pilot Study and Diagnostic Tests

A pilot study evaluated the validity, reliability, and clarity of content and timing of the research instrument with 50 students from similar backgrounds in the different groups of research participants, aged 10-18 years. After conducting reliability analysis using Cronbach ⍺, all questionnaire components had coefficients above 0.70, indicating strong internal consistency. The dimensionality of each scale was assessed using exploratory factor analysis, and construct validity, including convergent and discriminant validity, was examined using confirmatory factor analysis (CFA) with using SPSS AMOS software (IBM Corp). The scale’s average variance extracted (AVE) and composite reliability (CR) values were within the acceptable range for all structures, proving its validity. Variance inflation factor multicollinearity tests verified the dataset’s suitability for regression-based modeling. These tests showed that the independent variables were not highly correlated.

### Reporting Standards

This study followed the APA Journal Article Reporting Standards for Studies Using Structural Equation Modeling to provide comprehensive documentation of model construction, estimation methods, model fit indices, and mediation and moderation studies [83].

SEM was used to test the study’s hypotheses (H1, H2, and H3) and to estimate the direct and indirect correlations among variables for further statistical analysis. Furthermore, a moderated-mediation analysis was also performed for robust inferences. The study applied bootstrapping with 5000 resamples to establish the relevance of the indirect effects; the study accordingly performed the analysis. An interaction term was tested to assess whether AI-based feedback moderates the connection between gamified PE and cognitive performance. The relationship’s intensity and direction were considered. Model fit indices, including comparative fit index (CFI), Tucker-Lewis Index (TLI), root-mean-square error of approximation (RMSEA), and standardized root-mean-square residual, were within acceptable bounds, demonstrating model robustness. The relationships were exhaustively analyzed using SPSS Statistics version 26 (IBM Corp) and AMOS version 24 (IBM Corp) for all statistical methods.

Finally, this research relies on reliable statistical methods, measurement instruments, and rigorous sampling. By incorporating both technical and psychological components into educational interventions, such as gamified PE, Chinese secondary school students can gain a multifaceted understanding of how modern pedagogical strategies impact cognitive development. The stratified sample, high response rate, and advanced SEM methodologies make the findings reliable and accessible to a large audience.

### Ethical Considerations

This study was approved by the Research Ethics Committee of Kyonggi University, Suwon-si, Republic of Korea (Institutional Review Board no 12416). All procedures were conducted in accordance with institutional and national ethical guidelines. Written informed consent was obtained from all participants before data collection. For participants younger than18 years, written and oral consent was obtained from their parents or legal guardians. Trained facilitators assisted younger participants to ensure they clearly understood the survey items and participated voluntarily. All data were anonymized at the time of collection; no personal identifiers were recorded, and participants were assigned unique numeric codes for data handling and analysis. No images or other identifying materials of individual participants were collected or included in the paper. Therefore, no identifiable participant information is disclosed, and no additional consent was required for images.

## Results

Stratified random sampling was used to recruit students from various schools across the 3provinces, categorized by school type and geographic location. The study initially categorized schools by geography. Each stratum’s students were randomly selected. The study carefully balanced gender and grade levels for equitable sampling. PE instructors distributed questionnaires during class time with the support of administrators. Participation was voluntary, and students were given anonymity and confidentiality, which enhanced the legitimacy of the responses. School coordinators and field supervisors collaborated to gather data using standardized instruments over 2 months. Given the wide age range of participants, methodological and ethical adjustments were implemented for children in the 10- to 12-year age group, who may experience developmental difficulties in recalling intricate survey questionnaires. For this age group, the questionnaire was read aloud by trained facilitators in a structured manner. The facilitators read questions to the students individually, provided simplified explanations, and ensured that the students responded voluntarily without asking leading questions. This approach ensured the validity, accuracy, and adherence to ethical guidelines when using young respondents in psychological and educational research. The 13- to 18-year-old respondents were able to complete the questionnaire independently.

Table 2 provides the demographics of respondents to illuminate the study’s sample composition and contextualize the results. The sample comprised 1029 students from various educational institutions throughout China, ensuring a broad representation of student opinions and learning conditions. Male students comprised 47.60% (n=490) of the sample, while female students comprised 52.40% (n=539). The findings are more generalizable due to a gender-diverse participant pool and relatively equal distribution. Three age groups of students were 10-12, 13-15, and 16-18 years. The students included 438 (42.60%) aged 13-15 years, 310 (30.10%) aged 10-12 years, and 281 (27.30%) aged 16-18 years. This age group encompasses a broad range of developmental periods in middle and upper elementary and secondary school, making it crucial for gamified learning and cognitive growth. The survey found that 479 (46.60%) students assessed their academic performance as moderate, 305 (29.67%) as excellent, and 245 (23.83%) as low achievers. These distributions show how students from diverse academic backgrounds use gamified PE and AI-based feedback. Public schools educated 428 (41.60%) students, private schools 311 (30.20%), and semigovernment schools 290 (28.20%). This indicates a balanced representation across institutional types, as educational systems and funding may affect the integration of gamified PE and AI. The respondents were drawn from a variety of areas, including urban (366/1029, 35.60%), suburban (360/1029, 35%), and rural (303/1029, 29.40%) locations. Geographic diversity is considered when assessing the impact of educational infrastructure and technological accessibility on cognitive performance outcomes. As a result, the study also reveals regional variations in teaching and educational resources. Finally, 400 (38.90%) students reported high access to digital tools and platforms, 451 (43.90%) reported moderate access, and 178 (17.30%) reported limited access, a significant variable given this study’s focus on AI-based feedback. These results suggest that students’ technological readiness may influence or moderate the effectiveness of gamified learning approaches. Given the relatively high percentages of students with moderate to high technology access, most students appeared to have participated meaningfully using digital and AI-driven teaching resources.

Table 3 provides descriptive statistics, validity indicators, and internal reliability and stability for each of the study’s major latent variables, assessing the stability and internal consistency of the structural model’s constructs.

**Table 2:** Demographic characteristics of 1029 high school student respondents across Beijing, Shanghai, Chengdu, and Guangzhou, 2025.

Demographic variable and category			Value, n (%)
**Sex**			
	Male	490 (47.60)	
	Female	539 (52.40)	
**Age group**			
	10-12 years	310 (30.10)	
	13-15 years	438 (42.60)	
	16-18 years	281 (27.30)	
**Academic performance**			
	High achiever	305 (29.60)	
	Moderate achiever	479 (46.60)	
	Low achiever	245 (23.80)	
**School type**			
	Public	428 (41.60)	
	Private	311 (30.20)	
	Semigovernment	290 (28.20)	
**Geographic location**			
	Urban	366 (35.60)	
	Suburban	360 (35)	
	Rural	303 (29.40)	
**Technology access level**			
	High	400 (38.90)	
	Moderate	451 (43.80)	
	Low	178 (17.30)	

**Table 3:** Descriptive statistics, reliability, composite reliability, and average variance extracted for all study constructs based on 1029 student responses, 2025.

Variables	Value, mean (SD)	Cronbach α	CR^a^	AVE^b^
Cognitive performance	4.18 (0.72)	0.89	0.91	0.66
Gamified PE^c^ engagement	4.25 (0.68)	0.92	0.93	0.70
Physical activity enjoyment	4.12 (0.75)	0.87	0.89	0.64
Peer collaboration	4.05 (0.81)	0.86	0.88	0.61
Teacher support	4.1 (0.70)	0.91	0.92	0.69
Intrinsic motivation	4.21 (0.65)	0.90	0.91	0.68
AI^d^-based feedback	3.89 (0.79)	0.88	0.90	0.65

^a^CR: composite reliability.

^b^AVE: average variance extracted.

^c^PE: physical education.

^d^AI: artificial intelligence.

Cognitive performance, the study’s primary dependent measure, had a mean score of 4.18 (SD 0.72). With modest fluctuation, participants reported strong cognitive engagement and outcomes. The scale’s Cronbach ⍺ of 0.89 indicated high internal consistency. The CR was 0.91, and the AVE was 0.66, both exceeding the convergent validity criteria of 0.50. Scale elements adequately explained the hidden component. Gamified PE Engagement, a major independent variable, had the highest mean score (mean 4.25, SD 0.68), reflecting positive participant views of PE gamification. Cronbach ⍺ of 0.92, CR of 0.93, and AVE of 0.70 indicate the reliability of this scale. These statistics demonstrate that the gamified engagement measurement model is statistically valid and substantively relevant due to its strong internal cohesion. Another independent variable with a high mean score of 4.12 (SD 0.75) supported favorable participant attitudes toward PE. AVE 0.64, Cronbach ⍺ 0.87, and CR 0.89 all exceeded acceptable norms, indicating the construct’s internal reliability. Gamified activities motivated and engaged participants in PE. Peer collaboration also had strong psychometric features, with a mean of 4.05 (SD 0.81), CR=0.88, AVE=0.61, and Cronbach α=0.86. These findings support the favorable benefits of collaborative learning on academic success and demonstrate that the instrument effectively captures the social dynamics and cooperative learning aspects of PE. Teacher support was another major independent variable with high reliability and validity. A mean of 4.10 (SD 0.70), Cronbach ⍺ of 0.91, CR of 0.92, and AVE of 0.69 indicated a robust construct. These statistics highlight the role of instructors in designing, guiding, and supporting gamified education and physical learning, as well as cognitive outcomes and participant motivation. Most participants were motivated and self-driven in gamified learning contexts, with an average Intrinsic Motivation score of 4.21 (SD 0.65). This construct had an AVE of 0.68, a CR of 0.91, and a Cronbach ⍺ of 0.90. Self-determination theory suggests that intrinsic motivation is crucial for academic achievement, and these values support the statistical validity of the motivation construct. Finally, AI-based feedback had a lower mean of 3.89 (SD 0.79), suggesting a wider range of responses. However, Cronbach ⍺ scores of 0.88, CR 0.90, and AVE 0.65 indicate strong construct validity and internal consistency. Although experiences with AI feedback varied (possibly due to familiarity or availability), participants generally reported that it improved engagement and performance. Table 4 shows the item-level standardized loadings and metric invariance estimates.

**Table 4:** Item-level standardized loadings and metric invariance results for all constructs measured in 1029 high school students across 4 Chinese cities, 2025.

Variable and item		λ (standardized loading)	Cross-loading (max)	Mandarin translation ΔCFI^a^
**Cognitive performance (CP)**				
	CP1	0.78	0.12	0.003
	CP2	0.81	0.10	0.003
	CP3	0.83	0.09	0.003
	CP4	0.79	0.11	0.003
	CP5	0.82	0.10	0.003
**Gamified PE^b^ engagement (GPE)**				
	GPE1	0.85	0.11	0.004
	GPE2	0.88	0.10	0.004
	GPE3	0.86	0.12	0.004
	GPE4	0.84	0.09	0.004
	GPE5	0.87	0.10	0.004
**Physical activity enjoyment (PAE)**				
	PAE1	0.79	0.08	0.002
	PAE2	0.81	0.07	0.002
	PAE3	0.80	0.09	0.002
	PAE4	0.82	0.08	0.002
	PAE5	0.79	0.07	0.002
**Peer collaboration (PC)**				
	PC1	0.75	0.10	0.003
	PC2	0.78	0.11	0.003
	PC3	0.77	0.09	0.003
	PC4	0.76	0.08	0.003
	PC5	0.79	0.10	0.003
**Teacher support (TS)**				
	TS1	0.84	0.09	0.003
	TS2	0.87	0.08	0.003
	TS3	0.85	0.10	0.003
	TS4	0.86	0.09	0.003
	TS5	0.88	0.08	0.003
**Intrinsic motivation (IM)**				
	IM1	0.82	0.10	0.002
	IM2	0.85	0.11	0.002
	IM3	0.83	0.09	0.002
	IM4	0.81	0.08	0.002
	IM5	0.84	0.10	0.002
**AI^c^-based feedback (AIF)**				
	AIF1	0.80	0.09	0.003
	AIF2	0.83	0.10	0.003
	AIF3	0.81	0.08	0.003
	AIF4	0.82	0.09	0.003
	AIF5	0.84	0.10	0.003

^a^CFI: comparative fit index.

^b^PE: physical education.

^c^AI: artificial intelligence.

The study looked at item-level standardized loadings (λ) and cross-loadings for all latent constructs to ensure the study instrument was reliable and valid (refer to Table 5). The standardized loadings for each item were 0.75-0.88, indicating robust construct representation, while the cross-loadings were less than 0.12, indicating discriminant validity. The translation into Mandarin was tested for metric invariance, and all constructs had ΔCFI values <0.01, which indicates that the translated items perform the same as the original instrument. Table 5 provides a comparison of original CF and higher-order CFA models, which helps determine whether the measurement model in this research is legitimate.

**Table 5:** Comparison of original confirmatory factor analysis (CFA) and higher-order CFA models, including model fit indices, for constructs measured in 1029 high school students in Beijing, Shanghai, Chengdu, and Guangzhou, 2025.

Model	CFI^a^	TLI^b^	RMSEA^c^	SRMR^d^	*χ*² (*df*)	AIC^e^	BIC^f^
Original CFA^g^	0.961	0.953	0.042	0.038	2.85 (532)	2180.45	2245.67
Higher-order CFA (reduced model)	0.954	0.946	0.043	0.040	2.87 (534)	2145.32	2210.58

^a^CFI: comparative fit index.

^b^TLI: Tucker-Lewis Index.

^c^RMSEA: root-mean-square error of approximation.

^d^SRMR: standardized root-mean-square residual.

^e^AIC: Akaike information criterion

^f^BIC: Bayesian information criterion

^g^CFA: confirmatory factor analysis.

In a higher-order structural factor analysis (CFA), the study combined 2latent factors: intrinsic motivation and enjoyment of physical exercise, and cognitive performance and teamwork. Having a *χ*²/*df* ratio of 2.87 and good fit indices (CFI=0.954, TLI=0.946, RMSEA=0.043, and standardized root-mean-square residual=0.040), the higher-order model effectively fit the measurement model. The higher-order CFA has lower AIC (2145.32) and BIC (2210.58) than the original CFA, indicating that the reduced model is more parsimonious without losing theoretical or empirical validity. These results suggest that the dual-framework model (SDT + CLT) may retain construct validity and conceptual coherence with fewer latent variables.

The Fornell-Larcker criterion and the heterotrait-monotrait (HTMT) ratio of correlations were used to assess the discriminant validity of SEM. Table 6 provides their results.

**Table 6:** Fornell-Larcker criteria and the heterotrait-monotrait (HTMT) ratio assessing the discriminant validity of all constructs in 1029 high school students, 2025.

Constructs	Fornell-Larcker diagonal	HTMT^a^ ratios (max)
Cognitive performance	0.81	0.82
Gamified PE^b^	0.84	0.79
PE^b^ enjoyment	0.80	0.76
Peer collaboration	0.78	0.74
Teacher support	0.83	0.77
Motivation	0.82	0.81
AI^c^ feedback	0.81	0.80

^a^HTMT: heterotrait-monotrait.

^b^PE: physical education.

^c^AI: artificial intelligence.

The diagonal line shows the square root of each construct’s AVE compared to the interconstruct correlations, using Fornell-Larcker criteria. This method demonstrates discriminant validity when the square root of a concept’s AVE is greater than its highest correlation with any other construct. Cognitive performance’s Fornell-Larcker diagonal value of 0.81 is greater than its connections with gamified PE, motivation, and AI feedback. AI feedback (0.81), gamified PE (0.84), PE enjoyment (0.80), peer collaboration (0.78), teacher support (0.83), motivation (0.82), and AI Support (0.83) all have top diagonal values, indicating that each construct’s items are more strongly related to their latent variable than to any other. The HTMT ratio was used to enhance this investigation. PLS-SEM applies HTMT for a more sensitive and reliable discriminant validity test. Discriminant validity is good when HTMT values are <0.85. All HTMT scores in the results were between 0.74 and 0.82. For cognitive performance, none of the constructs has an HTMT ratio >0.82, which is sufficient. AI feedback (0.80), gamified PE (0.79), PE enjoyment (0.76), peer collaboration (0.74), teacher support (0.77), motivation (0.81), and AI support (0.79) all fall below the threshold, supporting the premise that each latent concept is empirically distinct. Table 7 shows the estimates of Harman single-factor and latent common method variance.

**Table 7:** Harman single-factor and latent common method variance (CMV) tests to evaluate potential survey bias in 1029 high school student responses, 2025.

Test type	No of factors extracted	Variance explained by first factor (%)	Total variance explained (%)	ΔCFI^a^ (with CMV^b^ factor)	Δ*χ*²^c^ (*df*)	*P* value	Conclusion
Harman single-factor test	7	28.6	72.4	—	—	—	No CMV concern (first factor<40%)
Latent common method factor test	7 + CMV	26.9	74.1	0.006	21.38 (1)	.09	No significant CMV effect (ΔCFI<0.01)

^a^CFI: common method variance.

^b^CMV: common method variance.

^c^Δ*χ*²: change in chi-square value.

Harman single-factor test and the latent CMV factor test were used to assess for self-reported data-related CMV. The unrotated factor analysis found 7 variables, although the first accounted for 28.6% of the variance (vs 40%). This indicates that no factor dominated the item-level covariance structure. This finding was confirmed by adding a latent CMV component to the measurement model. The model fit comparison showed no significant reduction in fit, even after controlling for method effects (ΔCFI=0.006 and Δ*χ*²_1_=21.38; *P*=.09). These findings strongly suggest that common method bias does not threaten the study’s validity and that discriminant integrity of latent constructs is preserved.

Table 8 provides the SEM findings, which strongly support the research’s theoretical approach. There is a positive relationship between gamified PE and students’ cognitive performance. Gamified PE may enhance students’ concentration, memory, and information-processing skills [84]. According to the engagement-learning paradigm, students learn meaningfully when they are emotionally, behaviorally, and cognitively involved [85]. Game elements, such as goal-setting, fast feedback, and reward systems, in PE sessions, may help children learn more effectively and improve cognitively [86]. Chaiyarat [87] and Aibar-Almazán et al [88] reported that gamification can make the classroom more dynamic and engaging, thereby enhancing students’ problem-solving and critical-thinking skills.

**Table 8:** Structural equation modeling (SEM) path estimates showing relationships among studied factors with standardized beta coefficients and significance levels, based on 1029 students, 2025.

Path	Estimates (β)	SE	*t* statistic	*P* value
Gamified PE^a^ → cognitive performance	.34^b^	0.05	6.80	<.001
Enjoyment → cognitive performance	.28^b^	0.06	4.67	<.001
Collaboration → cognitive performance	.26^b^	0.07	3.71	<.001
Teacher support → cognitive performance	.31^b^	0.06	5.17	<.001

^a^PE: physical education.

^b^*P*<.001 (1% significance level).

Teacher support is the second strongest predictor of students’ cognitive performance. This suggests that instructors must understand how to positively impact students’ cognitive growth through effective lesson preparation, positive reinforcement, and constructive feedback. Gamified and AI-feedback classrooms require teacher facilitation [89]. Teachers should motivate students by establishing a psychologically safe classroom, helping them grasp and implement challenging feedback, and scaffolding their learning to support their growth and development [90]. Cha et al [91] found that students feel more confident, engaged, and cognitively involved in classroom tasks, especially in active and digitally mediated settings, when they view their teachers as accessible, attentive, and helpful.

There is a positive relationship between physical activity enjoyment and students’ cognitive performance, which suggests that students who enjoy PE may focus better, experience lower cognitive stress, and feel emotionally good, which helps them learn. According to SDT, individuals are more engaged and learn more when they have a personal interest in the result [92]. Chen [93] found that children who enjoy physical activities are more willing to participate and better able to harness the psychological benefits of exercise, leading to improved classroom concentration, memory, and performance. Enjoyment enhances cognition in gamified environments by reducing performance anxiety and boosting self-confidence.

Peer collaboration and cognitive performance were significantly positively associated. Collaborating with peers during gamified PE classes boosts cognitive development. The focus on idea-sharing, collaborative problem-solving, and social and emotional support in peer learning enhances understanding and mental agility. This result is supported by Vygotsky’s sociocultural theory, which emphasizes the role of social interaction in internalizing information [94]. Qi and Derakhshan [95] found that physically active educational activities help students develop social and cognitive skills for academic success. Collaboration, negotiation, and peer feedback improve metacognition and learning.

The study analyzed cluster effects in Beijing, Shanghai, Chengdu, and Guangzhou using intraclass correlation coefficients (ρ) and design effects for each latent construct. The intraclass correlation coefficients ranged from 0.02 to 0.06, resulting in design effects of 7.18 to 19.90 with an average cluster size of 310 respondents per city (Table 9). After achieving the threshold (ρ>0.05), a multilevel structural equation model with random intercepts was used to review cognitive performance and AI-based feedback. There were minor differences in explained variance (Δ*R*²= +0.02 to +0.05) and standardized path coefficients (Δβ<.02) between single-level and multilevel estimations. The findings reveal that provincial clustering did not significantly affect gamified PE engagement, intrinsic motivation, and cognitive performance. Therefore, the estimation technique does not influence structural model robustness.

**Table 9:** Cluster diagnostics and multilevel structural equation modeling (SEM) robustness checks verifying the stability of results across school clusters in 1029 high school students, 2025.

Construct	Number of clusters (cities)	Average cluster size (n̄)	ICC^a^ (ρ)	Design effect=1+(n̄−1)ρ	Single-level β (gamified PE → cognitive performance)	Multilevel β (random intercept model)	Δβ^b^ (bias)	Δ*R*² (change in explained variance)
Cognitive performance (CP)	4 (Beijing, Shanghai, Chengdu, and Guangzhou)	310	0.05	16.45	.45	.43	−.02	0.04
Gamified PE^c^ engagement	4	310	0.04	13.36	.34	.33	−.01	0.03
Intrinsic motivation	4	310	0.03	10.27	.21 (indirect κ²=0.16)	.20	−.01	0.02
AI^d^-based feedback	4	310	0.06	19.9	.18 (interaction β)	.17	−.01	0.05
Teacher support	4	310	0.02	7.18	.31	.30	−.01	0.02
Peer collaboration	4	310	0.03	10.27	.26	.25	−.01	0.02

^a^ICC: intraclass correlation coefficient.

^b^Δβ: change in bias.

^c^PE: physical education.

^d^AI: artificial intelligence.

Table 10 assessed the impact of AI-based feedback on the model using effect-size indices (κ² and PM), conditional effects at ±1 SD, and Δ*R*². The mediation study revealed a medium-to-large effect size (κ²=0.19) and PM=0.36, suggesting that intrinsic motivation indirectly accounts for36% of the connection. Intrinsic motivation substantially influenced the link between gamified PE and cognitive performance (β=.23; *P*<.001). A moderated analysis indicated that AI-based feedback increased the positive correlation between gamified PE and cognitive performance (β=.17; *P*<.001). The correlation between AI feedback and gamified learning gains increased with complexity, with β=.13 at low levels (–1 SD), β=.20 at medium, and β=.28 at high levels (+1 SD). The inclusion of the interaction term raised the model’s explanatory power from *R*²=0.47 to *R*²=0.55, resulting in a Δ*R*² of 0.08, indicating that AI-based feedback explained an additional 8% of the variance in students’ cognitive performance.

**Table 10:** Moderation-mediation analysis showing effect sizes, conditional effects (±1 SD of artificial intelligence [AI]–based feedback), and ΔR² for relationships between gamified physical education (PE), intrinsic motivation, and cognitive performance in 1029 students, 2025.

Path	Estimate (β)	SE	*t* value	*P* value	LLCI^a^	ULCI^b^	κ²^c^	PM^d^	Conditional effect (–1 SD)^e^	Conditional effect (mean)^e^	Conditional effect (+1 SD)^e^	*R*²	Δ*R*²^f^
Mediation effect: gamified PE →intrinsic motivation →cognitive performance	.23^g^	0.04	5.75	<.001	0.15	0.32	0.19	.36	—	—	—	0.47	—
Moderation effect: gamified PE × AI feedback →cognitive performance	.17^g^	0.03	5.67	<.001	0.10	0.24	—	—	0.13	0.20	0.28	0.55	+0.08
Total effect: gamified PE →cognitive performance (including mediation and moderation)	.45^g^	0.05	9.00	<.001	0.35	0.55	—	—	—	—	—	—	—

^a^LLCI: lower limit CI.

^b^ULCI: upper limit CI.

^c^κ²: standardized indirect effect size.

^d^PM: proportion mediated.

^e^Conditional effects: changes in the slope of gamified PE → cognitive performance at low (–1 SD), mean, and high (+1 SD) levels of artificial intelligence (AI)–based feedback.

^f^Δ*R*²: 0.08, indicating an 8% increase in explained variance after counting the moderation term.

^g^*indicates a 1% significance level.

The quantity of AI feedback in gamified PE sessions affects student engagement and cognitive performance. Gamified PE improves cognitive function, and AI-based feedback enhances this impact. Children who participate in gamified PE and receive continuous, real-time AI-powered feedback are more likely to experience enhanced cognitive outcomes, including improved memory recall, attention, problem-solving, and mental engagement [96]. Feedback intervention theory suggests that timely and personalized feedback helps learners concentrate on task-related goals, self-regulate, and engage cognitively [97]. It may be challenging to obtain adaptive, data-driven insights in traditional PE settings, but AI-powered feedback is immediate, objective, and tailored. These systems, integrated into a gamified framework, make learning more engaging and challenging for students, providing explicit guidance on how to improve, which in turn fosters deeper thinking. According to Suresh Babu and Dhakshina Moorthy [98], gamification alone can boost student engagement. However, intelligent feedback systems can amplify these effects on cognition by influencing learner behavior and performance in real-time. The study’s findings support the use of technology-enhanced, customized learning environments in modern school design. Gamified education uses AI feedback as a cognitive support system to help students recall and apply what they have learned via hands-on, interactive activities [99]. CLT suggests that real-time AI feedback, which optimizes task difficulty and decreases ambiguity, helps learners focus on important cognitive activities. The moderating impact also affects curriculum and educational policy, suggesting that complex feedback systems are needed for gamified physical instruction to maximize cognitive outcomes. For gamified learning to be most effective, schools and instructors should use or invest in AI-powered solutions that tailor insights and feedback to each student’s unique profile and cognitive capabilities.

**Figure 2:**
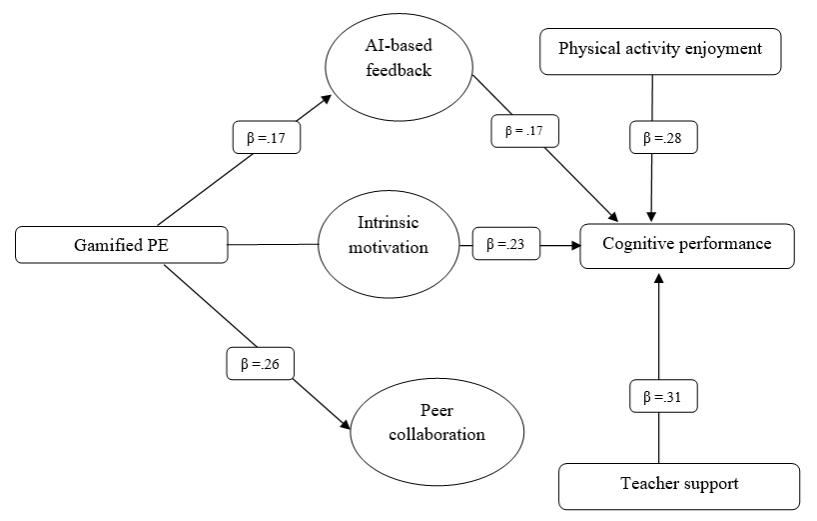
Structural model displaying standardized path coefficients for all hypothesized relationships among studied factors in 1029 high school students, 2025. Source: Author’s estimate.AI: artificial intelligence; PE: physical education.

Gamified PE improves cognitive function, and intrinsic motivation is a crucial psychological factor. Gamified PE enhances intrinsic motivation, leading to improved cognitive performance. The indirect effect explains most of the variance in cognitive performance, confirming that gamification’s structure and qualities are significant, but what counts most are learners’ psychological moods. Self-determination theory emphasizes relatedness, competence, and autonomy as components of intrinsic motivation, which is reflected in this mediation effect. Gamified learning environments enhance intrinsic interest and satisfaction by fostering autonomy through choice, competence through manageable tasks, and relatedness through peer collaboration [53]. Students who are genuinely motivated to study are more likely to use metacognitive skills, pay attention, and learn more deeply. Shalgimbekova et al [100] suggest that motivation moderates the link between instructional design and learning outcomes. This result highlights the role of motivation in mediating the pedagogical efficacy of innovative teaching methods. Even if gamified PE is fun and structured, internalizing values and desires drives cognitive growth. This study supports the idea that the motivating processes of instructional inputs are as essential as the inputs themselves in cognitive performance [101]. Gamification sets the scene, but intrinsic motivation propels cognitive functioning. This mediation strengthens Hypothesis H3, which states that intrinsic motivation mediates the relationship between gamified PE and cognitive performance. Gamification alone is ineffective; however, students’ natural incentive to participate and accomplish tasks substantially enhances outcomes, as evidenced by the considerable indirect path. This means that instructional designers, policymakers, and instructors should support children’s intrinsic drive to learn and incorporate engaging aspects into PE interventions to enhance academic cognition. Figure 2 shows the structural model with standardized path coefficients.

The significant first-order factor loadings (λ=0.65-0.88; *P*<.001) indicate high correlations between observable indicators and their latent components (refer to Table 11). The substantial second-order factor loadings (λ=0.71-0.84; *P*<.001) suggest that SDT and CLT collaborate to create a more sophisticated integrative model. The complete model suited the specified conceptual framework well, with CFI=0.957, TLI=0.949, and RMSEA=0.041.

**Table 11:** Structural equation modeling (SEM) results for the dual-framework model with standardized path coefficients in 1029 students, 2025.

Analysis	Statistic	*P* value
Second-order CFA^a^	Factor loadings: λ=0.65-0.88 (1st-order) and λ=0.71-0.84 (2nd-order)	<.001
Model fit indices	CFI^b^=0.957, TLI^c^=0.949, and RMSEA^d^=0.041	—^e^
Covariance	Covariance=0.42	<.001
Cross-loading check	Max cross-loading=0.25	—^e^
Latent interaction test	Δ*χ*²=4.12^f^	.04

^a^CFA: confirmatory factor analysis.

^b^CFI: comparative fit index.

^c^TLI: Tucker-Lewis Index.

^d^RMSEA: root-mean-square error of approximation.

^e^Not available.

^f^Indicates a 5% significance level.

Further, a significant association (covariance=0.42; *P*<.001) was found between intrinsic motivation and AI-based feedback, using a covariance approach. While preserving discriminant validity, this study supports the significant link between these factors. The measurement model was tested, and cross-loadings indicated that cognitive performance, intrinsic motivation, and AI-based feedback are empirically independent, supporting the structural coherence of the dual-framework SEM.

To determine whether AI-based feedback has context-specific effects, the study conducted multigroup moderation tests by level of technology access (high, moderate, and low) and location (urban vs rural; refer to Table 12).

**Table 12:** Multigroup moderation analysis examining differences in study relationships by urban vs rural location and technology access in 1029 high school students across 4 cities, 2025.

Path	Group	Sample size (n)	β estimate	SE	*t* value	*P* value	Δ*R*²^a^	f²^b^
Gamified PE^c^ → cognitive performance	Urban	366	.35^d^	0.06	5.83	<.001	0.05	0.16
Gamified PE → cognitive performance	Rural	303	.31^d^	0.07	4.43	<.001	0.05	0.15
Gamified PE → cognitive performance	High-tech access	400	.37^d^	0.05	7	<.001	0.07	0.18
Gamified PE → Cognitive Performance	Moderate tech access	451	.34^d^	.05	6.8	<.001	0.05	0.16
Gamified PE → cognitive performance	Low-tech access	178	.28^d^	0.06	4.67	<.001	0.04	0.14

^a^Δ*R*²: *P*<.05.

^b^f²: *P*<.05.

^c^PE: *P*<.05.

^d^Indicates a 1% significance level.

Table 12 shows a substantial influence of gamified PE on cognitive performance across all groups (β=.28-.37; *P*<.001). Impacts were higher for urban students and those with high technological access (β=.35 and .37, respectively) and lower for rural students and those with low access (β=.31 and .28, respectively). Moderation had medium to high impacts, with Δ*R*² values ranging from 0.04 to 0.07 and f² effect sizes from 0.14 to 0.18. These findings suggest that gamified PE interventions based on AI are generally effective, with urban students and those with greater technological access benefiting more cognitively.

## Discussion

### Principal Findings

This study examined how intrinsic motivation and AI-based feedback affect high school students’ cognitive performance after gamified PE. The first objective was to explore the effects of gamified PE and teacher support on students’ cognitive performance; statistically significant benefits were shown, confirming Hypothesis 1. The second goal was to assess how physical exercise enjoyment and peer collaboration affect cognitive performance, which was confirmed by the strong positive connections between these factors and cognitive performance, supporting Hypothesis 2. The findings supported the third aim, that is, determining whether gamified PE influences cognitive performance and whether intrinsic motivation mediated this impact. The positive effect of gamified PE on cognitive performance was partially mediated by intrinsic motivation, but AI-based feedback enhanced it, supporting Hypothesis 3. Gamified PE improves cognitive performance through motivational and technical processes, in line with the research’s goals and theoretical framework.

### Interpretation and Implications

Gamified PE improves cognitive function, suggesting that schools can create engaging, dynamic, and technologically advanced learning environments. Gamification boosts cognitive functioning by focusing students, simplifying problem-solving, and stimulating active involvement. Well-designed, gamified PE interventions may achieve these aims, supporting the view of Barz et al [102] that game-based learning may boost motivation and cognition. The SDT concept was reinforced by intrinsic motivation, which asserts that students who feel autonomous, competent, and socially engaged are more likely to participate in meaningful activities and achieve higher-order cognitive achievements. This suggests that gamified PE improves cognition and intrinsic motivation, which sustains engagement and learning [103]. Student involvement and academic success are highest in PE programs that incorporate enjoyable and independent activities. Social and instructional factors, including a friendly teacher, exercise, and teamwork, promote learning. Game-based and AI-supported learning benefit from human facilitation, although instructors’ guidance, encouragement, and scaffolding are still needed. Teamwork promotes collaboration, communication, and executive functioning, which may explain its cognitive advantages. These findings support more comprehensive theoretical frameworks that promote student-centered, socially engaged, and holistic learning environments [104]. AI-based feedback moderates the effect of adaptive, real-time recommendations on learning. AI-assisted strategy correction, refinement, and progressive advancement, delivered through rapid, tailored feedback, boosts cognitive performance beyond the reach of gamification. Recent AI-assisted learning experiments have shown potential for cognitive optimization and personalized skill improvement [105].

### Comparison With Existing Literature

This study builds on previous classroom gamification research. Gamification and AI feedback in PE can enhance cognitive performance, contrary to previous research on academic participants [106,107]. While gamification has been shown to improve motivation, little research has linked it to cognitive outcomes in PE [108,109]. These findings suggest that technology-driven feedback and compelling design may boost students’ cognitive performance. The study contributes to AI in education research by confirming previous classroom-based studies [110,111] that real-time adaptive feedback increases learning. This study combines SDT and CLT to advance theory. Gamified experiences increase learning via intrinsic motivation and cognitive load. These results provide a foundation for future research on integrated, technologically advanced, motivation-based educational interventions.

### Practical Implications

The findings suggest that AI-based feedback mechanisms could be beneficial to educational institutions, particularly in technologically advanced cities. To achieve this, AI-powered educational systems must be funded. These systems should have adaptive capabilities and age-appropriate user interfaces to provide children with timely, individualized feedback on their PE, behavioral engagement, and cognitive attention. Policy cooperation among Chinese education ministries, IT businesses, and AI research institutes may accelerate the development of educational systems tailored to the nation. To ensure that technology enhances human teaching, a national AI-in-education framework must guide deployment, data ethics, privacy protection, and teacher capacity-building. Pilot studies in schools with suitable digital infrastructure may examine the effects of AI-enhanced, gamified learning on academic achievement. Since public and semigovernment institutions had larger performance disparities, the findings imply a shift toward socially and motivationally enhanced learning. Policies should prioritize training PE instructors to create welcoming, inclusive, and inspirational learning environments. To stimulate cognitive development through intrinsic motivation and peer collaboration, schools must prioritize students’ mental health, incorporate team-building activities, and use inclusive instructional techniques that cater to their unique social identities and needs. Teacher performance measurements should incorporate student-centered learning, emotional support, and inclusive engagement as part of education reforms. Physical exercise, reflection, feedback, and cognitive challenge must be balanced in gamified learning settings that promote collaboration and active learning.

### Limitations

This study has made many essential contributions. However, there are some limitations. Although the research used a large and diverse sample from 4 metropolitan cities, its cross-sectional design limits causal inferences. SEM and bootstrapping provide more compelling findings; however, experimental or longitudinal designs are necessary to determine the long-term impact of gamified PE and AI feedback on cognitive function. Second, self-report assessments may be biased by social desirability or cognitive misunderstanding, even when administered under supervision to children aged 10-12 years. Future studies could triangulate findings and improve measurement validity by using multi-informant data, such as behavioral observations or teacher ratings. The generalizability of the results is another issue. The sample included students from urban and suburban public, private, and semigovernment schools, but not rural or low-tech institutions. This raises the question of how AI-based gamified systems can effectively serve diverse socioeconomic and geographical conditions. Future research should examine how infrastructural variations affect such efforts and the digital divide. Although this study focused on cognitive function, it did not examine emotional regulation, academic resilience, or physical health. Future multidimensional research should examine how gamified and AI-supported instructional techniques affect broader student capacities. This study focused on AI feedback as a moderator and intrinsic motivation as a mediator. Other factors, such as self-efficacy, goal orientation, and support from family and friends, influence students’ attitudes toward gamified learning. Future studies should incorporate additional mediators and moderators to gain a deeper understanding of the complex interplay among human, systemic, and technological factors. In conclusion, qualitative research methods, such as student interviews or classroom ethnographies, can complement quantitative approaches by providing contextual insights into learners’ experiences and preferences. By addressing these limitations, future research may enhance the theoretical and practical understanding of how gamified, technology-enhanced education can improve student learning and growth.

### Conclusions

The study concludes that Gamified PE with intrinsic motivation and AI-based feedback improves high school students’ cognitive performance. These findings demonstrate that technology-based student-centered PE programs improve engagement, motivation, and cognition. Gamification and AI in education may improve students’ overall development and academic and social results outside of the classroom. Future research should examine the long-term impacts, demographic factors, and technological developments to enhance the cognitive and motivational benefits of school-based treatments. To capitalize on the full potential of gamified PE for cognitive development, policymakers in education must institutionalize gamified pedagogy into the national PE curriculum. Schools should update their PE curriculum to highlight the connection between physical health and emotional and intellectual development. The curriculum should incorporate more structured, game-based learning methods that enhance cognitive abilities. In PE, goal-setting, point-scoring, and challenge-based learning help students concentrate, recall, and solve issues. The Ministry of Education, along with state and regional education bureaus, should educate PE teachers in gamified teaching methods to meet pedagogical criteria, engage students, and measure academic achievement.

This study advances the literature by introducing a technology-driven model of PE that combines gamification with AI-based feedback to enhance students’ cognitive abilities. This study demonstrates the synergistic effects of gamification, intrinsic motivation, and AI-supported feedback on cognitive development, in contrast to previous research that has primarily focused on conventional PE or singular teaching methodologies. This study contributes to the existing literature on digital and AI-enhanced education by using a substantial, multicity sample and SEM to demonstrate the validity of these connections. The findings have substantial implications for educational institutions, educators, and policymakers, suggesting that interactive, AI-driven PE programs can effectively enhance student engagement, learning processes, and cognitive performance in genuine educational environments.

## Data Availability

The dataset generated and analyzed during this study are not publicly available due to privacy protections for minors and institutional restrictions. Deidentified data may be made available upon reasonable request to the corresponding author, subject to ethical approval.

## Abbreviations

AI: artificial intelligence
AVE: average variance extracted
CFA: confirmatory factor analysis
CFI: comparative fit index
CLT: cognitive load theory
CR: composite reliability
HTMT: heterotrait-monotrait
PE: physical education
RMSEA: root-mean-square error of approximation
SDT: self-determination theory
SEM: structural equation modeling
TLI: Tucker-Lewis Index
